# Repercussions of the COVID- 19 pandemic on maternal and congenital syphilis in South Brazil: a time series analysis 2010-2022

**DOI:** 10.1186/s12879-025-10901-x

**Published:** 2025-04-15

**Authors:** Fernando Echegaray, Christopher J. Hernandez, Kavya G. Sundar, Lanbo Z. Yang, Mary Catherine Cambou, Eddy R. Segura, Marineide Gonçalves de Melo, Breno Riegel Santos, Ivana Rosângela dos Santos Varella, Karin Nielsen-Saines

**Affiliations:** 1 UCLA David Geffen School of Medicine, Los Angeles, CA USA; 2 Department of Obstetrics and Gynecology, SUNY Downstate Health Sciences University, Brooklyn, NY USA; 3 Department of Obstetrics and Gynecology, Tulane University School of Medicine, New Orleans, LA USA; 4 Department of Medicine, Division of Infectious Diseases, UCLA David Geffen School of Medicine, Los Angeles, CA USA; 5 Facultad de Ciencias de la Salud, Universidad Científica del Sur, Lima, Peru; 6 Department of Infectious Disease, Hospital Nossa Senhora da Conceição, Sistema Único de Saúde, Porto Alegre, Brazil; 7 Department of Epidemiology and Public Health, Hospital Nossa Senhora da Conceição, Sistema Único de Saúde, Porto Alegre, Brazil; 8 Department of Pediatrics, Division of Pediatric Infectious Diseases, UCLA David Geffen School of Medicine, Los Angeles, CA USA

**Keywords:** Gestational syphilis, Congenital syphilis in southern Brazil, Holt-Winters time series, COVID- 19 pandemic, Congenital syphilis trends, Syphilis adverse birth outcomes

## Abstract

**Background:**

The global increase in maternal and congenital syphilis cases over the past decade has been substantial. In south Brazil, preexisting maternal and congenital syphilis epidemics have been worsened since the onset of COVID- 19. We evaluated the impact of the COVID- 19 pandemic on the epidemiological trends of maternal and congenital syphilis in Porto Alegre, Brazil.

**Methods:**

We conducted a retrospective review of hospital records from a large public hospital network, covering the full period of January 1, 2010, to December 31, 2022. Based on historical maternal syphilis and congenital syphilis cases from 2010 to 2019, a Holt-Winters seasonal forecasting model was used to predict maternal syphilis prevalence from 2020 to 2022. A subanalysis of total births, corresponding maternal syphilis prevalence, congenital syphilis cases and infant outcomes was performed for 2017 to 2022 to take a closer look at the years preceding and following the pandemic onset. The diagnoses of maternal and congenital syphilis were determined according to the Brazilian Ministry of Health guidelines.

**Results:**

The Holt-Winters model predicted relatively stable maternal syphilis prevalence from 2020 to 2022. In contrast, the observed prevalence at delivery was higher than predicted: in 2021 and 2022, forecasting predicted average yearly prevalences of 8.7% and 8.8%, while observed prevalences were 12.5.% and 10.3%. Total births throughout 2017–2022 remained stable with mild decline in the pandemic period. Total maternal syphilis prevalence did not change from 11.1% in 2019 to 2020. However, the percentage of patients diagnosed with syphilis at delivery increased from 14% in 2019 to 65% in 2020. A statistically significant increase in total maternal syphilis prevalence from 11.1% in 2020 to 14.8% in 2021, *p* ≤ 0.05 was noted. Congenital syphilis diagnoses decreased from 57 to 24% during the pre-pandemic period and increased to 27% in 2022. The incidence of fetal demise in syphilis-positive patients declined throughout the study period from 14% to 6.9% equating roughly 30 cases per year.

**Conclusion:**

The COVID- 19 pandemic was a significant setback in recent progress made toward the control of maternal and congenital syphilis in south Brazil. Public health strategies should prioritize reinstatement of interventions for prevention of gestational syphilis.

## Introduction

Untreated maternal syphilis infection is associated with serious adverse outcomes, including preterm delivery, prolonged neonatal intensive care unit admission, spontaneous abortion, and stillbirth [1–5]. Case fatality estimates range from 31 to 65% [6–8]. Neonatal diagnosis is challenging, with only approximately 15% of newborns displaying symptoms at birth [9]. Undetected cases can lead to irreversible complications such as dental abnormalities, hard palate defects, nasal cartilage destruction, joint swelling, and deafness [10]. Vertical transmission rates of syphilis can decrease from nearly 100% in pregnant patients with untreated primary syphilis to 1–2% with proper penicillin treatment during pregnancy, highlighting the critical need for improved detection, treatment, and follow-up [11].

In the past decade, syphilis infection during pregnancy and congenital transmission have reemerged as significant public health challenges globally [12]. Recent global estimates have indicated a reduction in congenital syphilis cases from 749,000 in 2012 to 661,000 in 2016 [9, 13]. However, in the United States alone, maternal syphilis rates tripled between 2016 and 2022 [14]. In Brazil, rates of maternal syphilis increased more than six-fold from 2008 to 2018 [15]. International initiatives have been directed toward reducing maternal syphilis, however, congenital syphilis rates remain well above the World Health Organization (WHO) 2007 elimination target of 50 cases per 100,000 live births [16, 17].

The COVID- 19 pandemic has exacerbated these challenges. In Brazil, maternal syphilis remains prevalent, resulting in a consistently high incidence of congenital syphilis despite comprehensive healthcare access. Epidemiological reports indicate that from 2012 to 2022, syphilis prevalence in the general adult population in Brazil increased annually, with the exception of 2020 [18]. This anomaly is attributed to reduced diagnostic capacity during the COVID- 19 pandemic. The COVID- 19 pandemic led to a dramatic decline in preventative and treatment services provided by the Brazilian Unified Health System (SUS) [19]. Maternal syphilis prevalence has also been rising steadily each year. In 2022, the maternal syphilis prevalence reached 32.4 cases per 1,000 live births in all of Brazil, representing a 15.5% increase from the previous year [18]. Similarly, congenital syphilis cases increased by 19.1% from 2017 to 2022 [18].

This study aimed to analyze recent trends in maternal syphilis in a representative public health network in South Brazil and its impact on congenital syphilis and infant birth outcomes during the first three years of the COVID- 19 pandemic. Our goal was to enhance the understanding of maternal and child health trajectories in regard to syphilis diagnoses in the peripartum period amid unprecedented public health challenges. By comparing data from before and after the pandemic, we aimed to evaluate the impact of the pandemic on maternal and congenital syphilis resulting from disruptions in healthcare access in the region of Porto Alegre. Given the location of our institution in the capital of the southernmost state of Brazil, Rio Grande do Sul, the findings of this study shed light on the impact of the COVID- 19 pandemic on maternal and congenital syphilis in this region.

## Methods

This retrospective cohort analysis utilized data extracted from electronic health records at a large, tertiary healthcare network in Porto Alegre, Brazil and was performed from November 2023 to July 2024. The study was approved by the local institutional review board at the Grupo Hospitalar Conceição, Sistema Único de Saúde, Porto Alegre, Brazil and the University of California Los Angeles institutional review board. The study spans data from January 1, 2010, to December 31, 2022, encompassing both pre-pandemic and pandemic periods.

By retrospectively comparing recorded data from before and after the pandemic in an otherwise stable group of patients with documented deliveries, we aimed to evaluate the impact of the pandemic on maternal and congenital syphilis resulting from disruptions in healthcare access caused by the COVID- 19 spread and lockdowns.

### Data collection and definitions

Total monthly and yearly births were calculated using institutional monthly birth reports. We evaluated pregnancy endpoints, including live birth deliveries and fetal losses due to distinct circumstances, as well as stillbirths, and twin or triplet gestations. These numbers were used as denominators to subsequently determine the prevalence of syphilis detected during gestation, the prevalence of syphilis detected until delivery, congenital syphilis transmission events, and total maternal syphilis prevalence which are further defined below.

Syphilis status during gestation (SSg) data were collected from gestational syphilis prenatal care institutional records. The criteria for a positive SSg were defined as: (1) a concurrent positive result on a rapid treponemal immunoassay and a VDRL (Venereal Disease Research Laboratory) test or (2) a positive result on a VDRL test with corresponding clinical assessment (only for years prior to 2019 due to lack of rapid treponemal immunoassay availability). Pregnant patients with documentation of adequate syphilis treatment in the past and for whom reinfection in the current pregnancy was ruled out were excluded from this definition.

Syphilis status at delivery (SSd) was recorded for all individuals who presented for labor and delivery services, where universal screening for HIV and syphilis is mandated according to the institutional protocol. SSd was determined using a combination of rapid treponemal immunoassays, VDRL testing, and clinical assessment, adhering to the diagnostic algorithms of the Brazilian Ministry of Health [20, 21]. The Brazilian universal healthcare system, Sistema Único de Saúde (SUS), supplied rapid testing kits used for initial screenings. The criteria for a positive SSd were defined as: (1) a concurrent positive result on a rapid treponemal immunoassay and a VDRL test or (2) a positive result on a VDRL test with corresponding clinical assessment (only for years prior to 2019 due to lack of rapid treponemal immunoassay availability) or a (3) indeterminate maternal treponemal or VDRL test results with subsequent confirmation of vertical transmission of syphilis. Pregnant patients with documentation of adequate syphilis treatment in the past and for whom reinfection in the current pregnancy was ruled out were excluded from this definition.

Congenital syphilis transmission (CST) events were determined using the Brazilian Ministry of Health and Center for Disease Control (CDC) congenital syphilis 2015 case definition [22]. The criteria for a CST event were defined as demonstration of Treponema pallidum by: (1) darkfield microscopy of lesions, body fluids, or neonatal discharge; (2) polymerase chain reaction or other equivalent direct molecular methods of lesions, neonatal nasal discharge, placenta, umbilical cord, or autopsy material, or (3) immunohistochemistry, or special stains (e.g., silver staining) of specimens from lesions, placenta, umbilical cord, or autopsy material. A probable case condition was considered when an infant’s mother had untreated or inadequately treated syphilis at delivery, regardless of signs or subsequent testing in the infant, or if an infant or child had a reactive non-treponemal test for syphilis (VDRL, RPR, or equivalent serologic methods) and any one of the following: 1. Untreated or inadequate maternal syphilis in mother; 2. Any evidence of congenital syphilis on physical examination; 3. Any evidence of congenital syphilis on radiographs of long bones; 4. Reactive CSF VDRL test; 5. In a nontraumatic lumbar puncture, an elevated CSF leukocyte count or protein (without other cause) detected.

The total maternal syphilis prevalence (TMSP) was defined as the total number of patients who had a positive SSg or SSd for a certain year divided by the total number of births at the institution for that same year. Duplicate patients identified in prenatal care and during delivery were counted as one positive case. Patients who were identified in prenatal care but did not deliver at our institution were included as one positive SSg case.

### Statistical approach

Using Stata (version 17.0; StataCorp LLC, College Station, TX), a Holt-Winters (HW) seasonal forecasting model was used to predict syphilis prevalence after the pandemic onset [23]. Monthly SSd rates from January 1, 2010, to December 31, 2019, were used to generate a smooth forecasting prediction for monthly syphilis rates from January 1, 2020, to December 31, 2022. Smoothing parameters (0.5 α), (0.1 β), and (0.1 γ) provided the lowest root mean squared error of 1.7. To determine any significant deviations indicative of the pandemic's influence, the forecasts were compared to the observed syphilis rates during the pandemic using GraphPad Prism software (version 10.2.3 for Mac. GraphPad Software, Boston, Massachusetts USA). Ninety-five [95%] confidence intervals were calculated using Stata and displayed using Prism.

Yearly births and TMSP (using both SSg and SSd) were calculated using Stata and Prism. Fraction of total estimates for maternal syphilis prevalence were performed using the Wilson/Brown method with a 95% confidence interval. Yearly congenital syphilis transmission rates and associated infant birth outcomes were extracted using Stata, and the data were displayed using Prism.

## Results

**Fig. 1:**
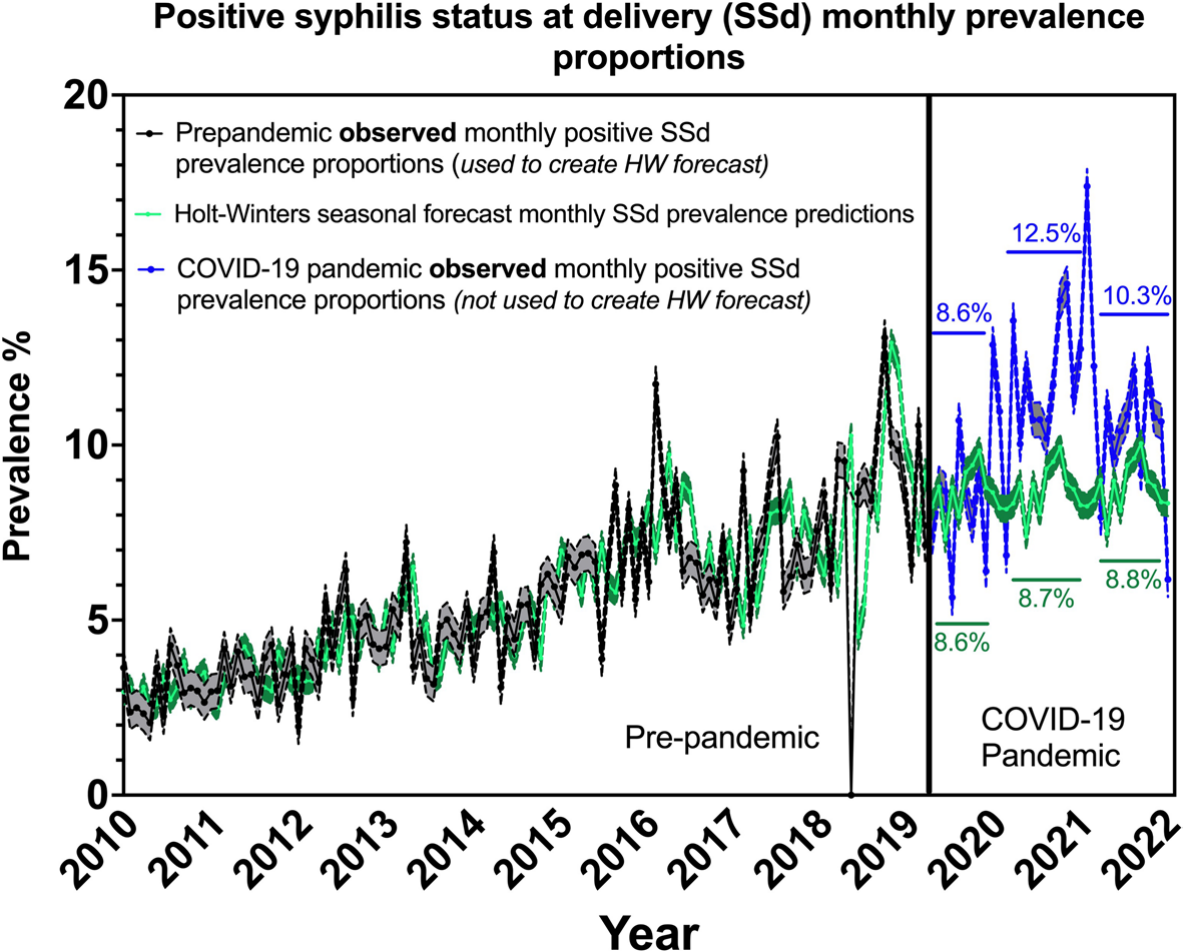
Positive syphilis status at delivery monthly prevalence proportions. Prepandemic observed monthly positive SSd rates used in forecasts are represented by black dots and connected with black lines with gray upper and lower 95% confidence intervals on the left panel. Holt-Winters seasonal monthly predictions (for the prepandemic period extending into the COVID- 19 pandemic period) are shown as lime green dots and connecting lime green lines with dark green upper and lower 95% confidence intervals. The observed monthly rates for the COVID- 19 pandemic period not used in the monthly forecasts are represented by blue circles and blue connecting lines with gray upper and lower 95% confidence intervals on the right panel

The number of pregnant patients with a positive SSd steadily increased over the past decade at our institution in south Brazil, as shown in black in Fig. 1. Before 2015, it was unusual for the monthly positive SSd prevalence to exceed 5%, an already very elevated syphilis prevalence. After 2015, except for a few outlier months, the prevalence of positive SSd did not decrease to less than 5%. Monthly peaks reached a positive SSd prevalence as high as 12 to 13% in 2016 and 2019. Overall, the trend showed a continuous increase with monthly seasonal variations.

The HW seasonal forecast, as shown in green in Fig. 1, accurately fit the observed monthly positive SSd prevalence for the pre-pandemic period. After the onset of the pandemic, we observed that the forecast prediction displayed fluctuating monthly variations of approximately 8 to 10% in positive SSd prevalence.

The observed monthly prevalence, as shown in blue in Fig. 1, demonstrated a drastic difference between the forecast prediction and reality. The prevalence during the pandemic period was significantly greater than what was predicted by the forecast model. HW forecast predictions revealed yearly average prevalences of 8.6%, 8.7%, and 8.8% in 2020, 2021, and 2022, respectively. However, the observed yearly average positive SSd prevalences were 8.6% for 2020, 12.5% for 2021, and 10.3% for 2022. In December of 2021, the forecast predicted a monthly positive SSd prevalence of 8.3% while we observed a staggering 17.4% at our institution.

**Fig. 2:**
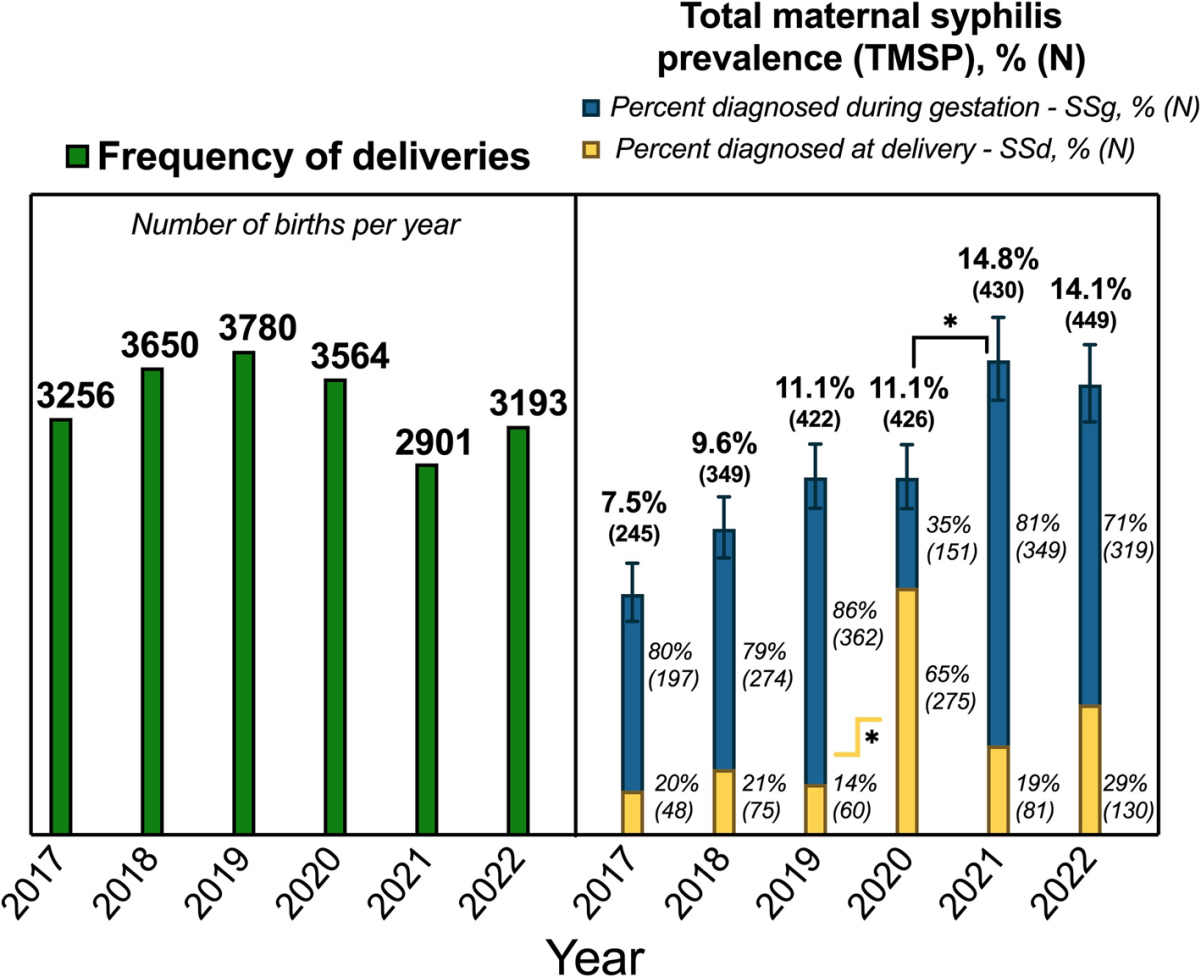
Total deliveries and total maternal syphilis prevalence per year from 2017–2022. The bar height in green represents the total number of births per year in the left panel. The bar height in the right panel represents the total maternal syphilis prevalence for that year with 95% confidence intervals. The blue section of the bar represents the percentage of patients diagnosed with syphilis during prenatal care. The yellow section of the bar represents the percentage of patients diagnosed with syphilis at delivery. *Indicates statistically significant differences (Wilson/Brown *p* ≤ 0.05) between total maternal syphilis prevalence proportions between years

Despite the increase in positive SSd prevalence, the total number of births within our health system remained relatively steady throughout the study period from 2017 to 2022, with approximately 3400 births per year (as shown in Fig. 2), notwithstanding potential challenges posed by the SARS-CoV- 2 pandemic. Regardless of this overall stability, a slight decrease in birth rate (2901) was noted in 2021. However, this decline did not correspond with the trend in total maternal syphilis prevalence (TMSP), which continued to rise steadily. Using the Wilson/Brown method, there was a statistically significant increase in TMSP from 11.1% to 14.8% between 2020 and 2021, *p* ≤ 0.05. Notably, the percentage of patients with syphilis diagnosed at delivery increased nearly five-fold to 65% in 2020 compared to 14% in 2019, *p* ≤ 0.05 (Fig. 2).

**Fig. 3:**
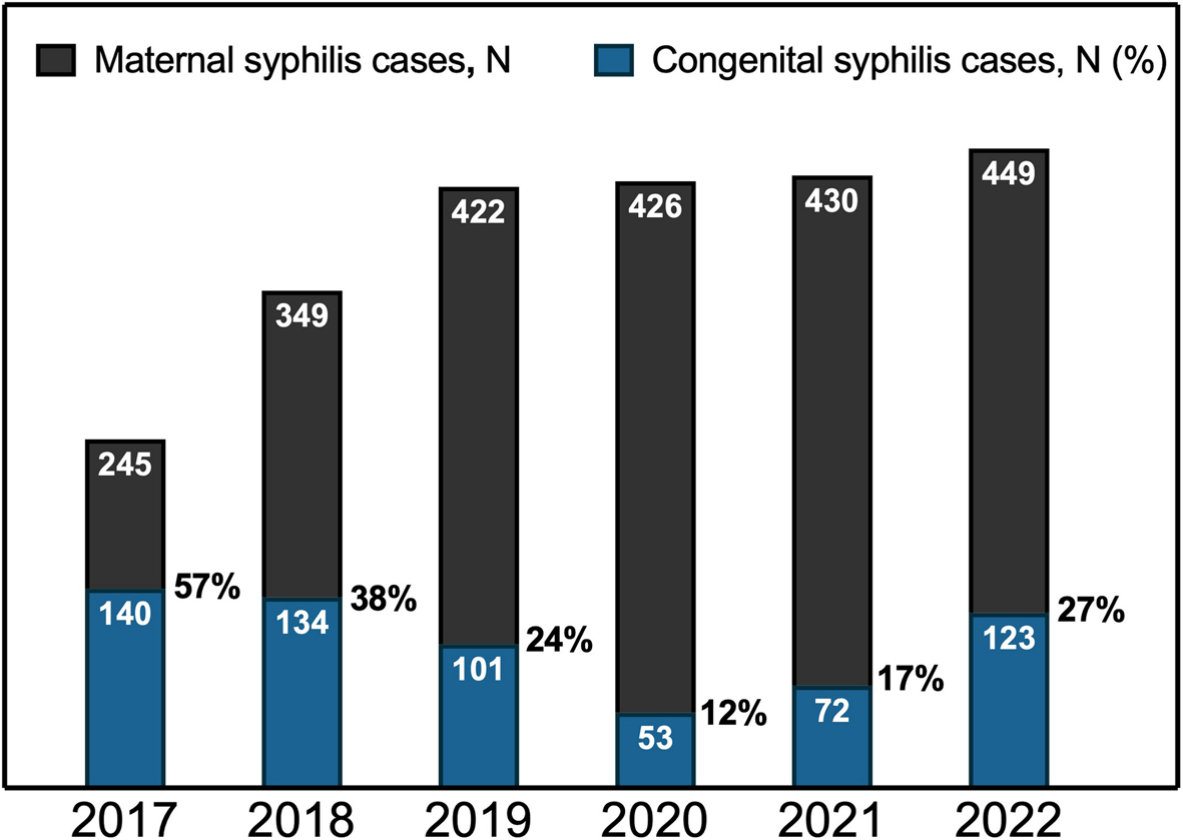
Congenital syphilis cases in relation to gestational syphilis 2017–2022. The total black bar height represents the total maternal syphilis cases for that year. The blue bar height represents the total number of congenital syphilis transmission events that occurred during that year. The percentage next to the blue bar represents the incidence of congenital syphilis transmission

Before the onset of the COVID- 19 pandemic, there were indications of slight improvement in the incidence of congenital syphilis at our institution, as shown in Fig. 3. In 2017, congenital syphilis cases occurred in approximately 57% of confirmed maternal syphilis cases. This number decreased to 24% in 2019. Of note, in 2020 the CST rate was the lowest observed (12%), most likely due to decreased detection capacity secondary to COVID- 19 pandemic healthcare disruptions. However, since the beginning of the pandemic, an increase in the number of congenital syphilis cases was noted. Despite initial progress, by 2022, rates of congenital syphilis approached 27%, once again showing a reemergence of the problem.

**Fig. 4:**
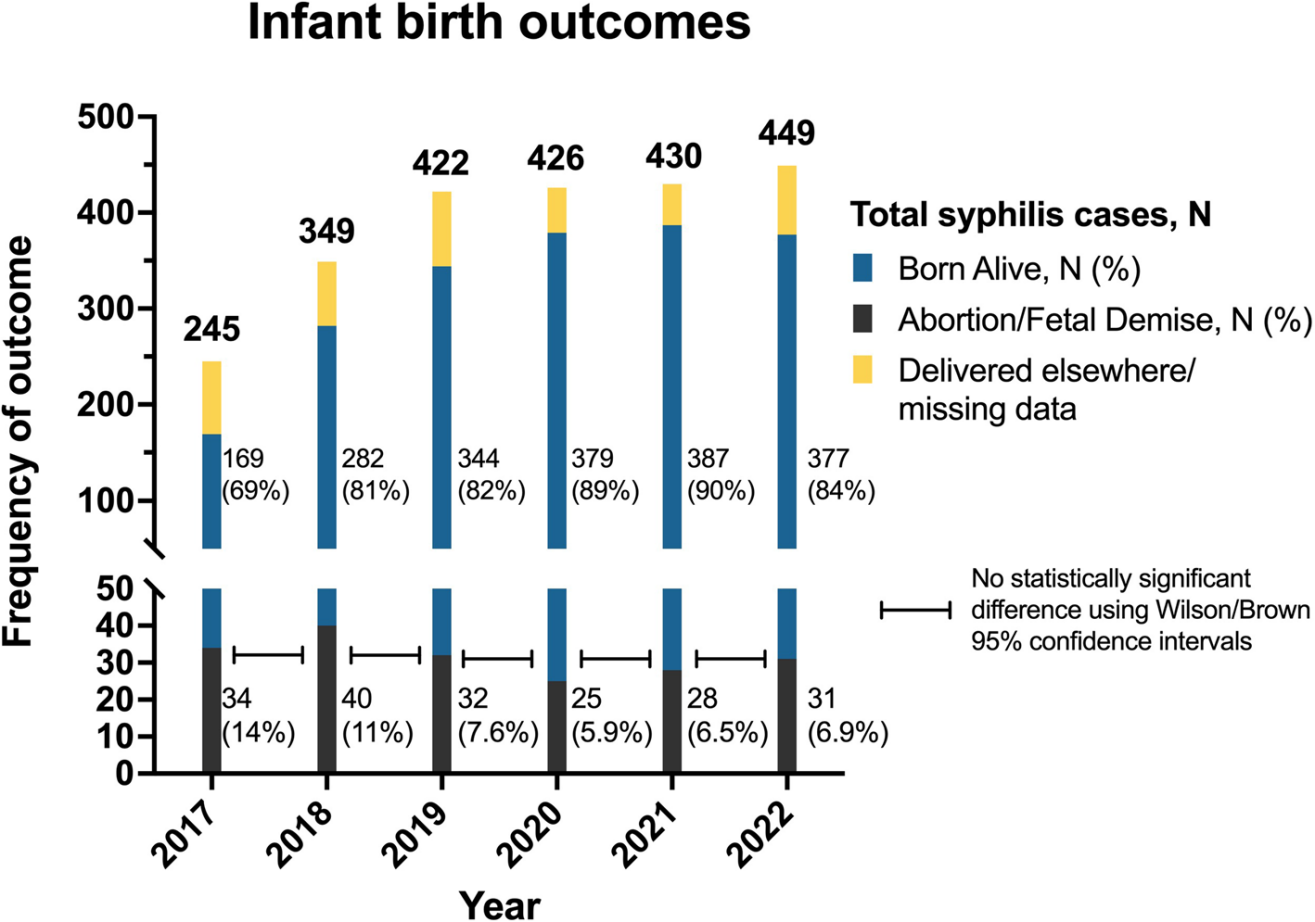
Gestational syphilis infant birth outcomes. The total bar height represents the total maternal syphilis cases for that year. The blue bars represent the total number of infants born alive to syphilis positive patients. The black bars represent the total number of abortions/fetal deaths in syphilis positive patients. The yellow section represents missing data, or patients who delivered elsewhere. The black interconnecting bars represent no statistically significant difference between infant birth outcome rates for each year when using the Wilson/Brown method with 95% confidence intervals

Throughout the period studied, the rate of adverse birth outcomes in pregnancies affected by syphilis declined, with fetal losses (miscarriages and stillbirths) decreasing from 14% in 2017 to 6.9% in 2022, as shown in Fig. 4. No worsening of adverse birth outcomes was noted following pandemic onset. No statistically significant difference was observed between infant birth outcome rates. However, the raw number of miscarriages and fetal deaths in syphilis-positive patients remained stable at approximately 30 cases per year.

## Discussion

In 2021, the Brazilian Ministry of Health reported that among state capitals, Porto Alegre had the second highest rate (6.15%) of maternal syphilis cases detected during gestation but the country’s highest incidence (3.95%) of congenital syphilis cases [18]. Lack of prenatal care, ineffective penicillin treatment, and partner reinfection have been shown to be associated with an increased risk of congenital syphilis cases in Porto Alegre [24]. Additionally, widespread undertreatment occurs in patients with low VDRL titers [25]. Given the location of our institution in the capital of the southernmost state of Brazil, Rio Grande do Sul, the findings of this study shed light on the impact of the COVID- 19 pandemic on maternal and congenital syphilis in this region of Porto Alegre. By comparing data before and after the onset of COVID- 19, this study assesses the effects of an unprecedented pandemic on the epidemiology course of co-existing infectious disease epidemics in this southernmost region of Brazil.

Using only the SSd from 2010 to 2019, we created an HW seasonal forecasting model to predict the prevalence of maternal syphilis during the pandemic, and the observed prevalence proportions since pandemic onset starkly diverged from the anticipated trends. This revealed a discrepancy that underscores the unpredictability of disease dynamics during times of crisis. Moreover, the consistent number of births throughout the study period shows that a resilient healthcare infrastructure is essential for ensuring access to obstetric services despite external challenges.

We observed a sharp increase in the proportion of cases of maternal syphilis diagnosed at the time of labor and delivery in the first year of the pandemic, a nearly five-fold increase compared to prior years, which underscores that prenatal care suffered dramatically in the pandemic lockdown phase [19]. The rate of maternal syphilis detected at delivery in 2020 likely resulted from a combination of factors during the pandemic, including reduced access to care, limited testing capacity, and decreased mobility resulting from lockdown measures [26]. A number of patients may not have been effectively diagnosed during this critical time. The lack of appropriate identification of maternal syphilis cases during gestation translates into an increasing number of congenital syphilis cases observed in 2021 and 2022. Congenital syphilis is already a public health challenge in this area and the COVID- 19 pandemic added a completely new layer of complexity in attempting to reduce congenital syphilis rates.

In the years preceding the pandemic, congenital syphilis rates were successfully diminishing at our institution. In 2016, following the WHO Global Health Sector Strategy on Sexually Transmitted Infections and Brazil's declaration of a syphilis epidemic, the Brazilian government launched the"Syphilis No"public health project, which employed nationwide mass communication to disseminate information on testing, prevention, and treatment [27]. This national public health initiative focused on improving education to key populations may have contributed to the observed results [27]. Following the COVID- 19 pandemic lockdowns in 2020, congenital syphilis rates decreased to artificially low rates due to decreased testing capacity in an overburdened healthcare system [26]. However, in 2021 and 2022, incidence rates increased to numbers almost as high as before the beginning of the “Syphilis No” project. This shows how prior successful efforts to reduce this epidemic, have been impaired by this public health crisis.

Lastly, although the raw number of pregnancy losses associated with maternal syphilis remained relatively stable throughout the study period, the percentage of observed miscarriages/stillbirths in mothers with gestational syphilis declined from 14 to 7%. Nevertheless, it is crucial to underscore the harmful impact of syphilis infection during pregnancy on maternal and neonatal health outcomes [2, 4]. Even though the yearly rates may not have worsened dramatically, they remain unacceptably high from a public health standpoint.

The disruptions in healthcare caused by the COVID- 19 pandemic have led to an increased prevalence of syphilis in pregnant patients and their newborns. The elevated rates of gestational and congenital syphilis highlight the need for intensified efforts to enhance access to prenatal care, promote comprehensive screening protocols, and ensure prompt treatment of pregnant patients infected with syphilis regardless of emerging epidemics. Public health strategies should refocus on addressing these persistent infections, as they have not disappeared and continue to pose significant risks to maternal and infant health.

### Strengths and limitations

The most notable strength of our study was the robust sample size with data from more than 75,000 deliveries over a 15-year period. Another strength was the ability to quantify and separate syphilis status during gestation and syphilis status at delivery since estimates tend to underestimate maternal syphilis prevalences due to preferential reporting of delivery data. Another strength was the ability to produce a HW seasonal forecast using monthly proportions that allowed significant prediction robustness due to the use of granular data.

However, while the nature of our dataset permitted a robust comparative analysis of the effect of the COVID- 19 pandemic on the epidemiology of maternal and congenital syphilis, there are limitations. In particular, given the extensive volume of data required for monthly syphilis rate abstraction in a decade-long analysis, only the syphilis status at delivery was used to generate the HW forecasting model. This approach may have underestimated the observed rates since prenatal gestational syphilis records were not included in this portion of the analysis.

## Conclusion

By comparing data before and after the pandemic to assess the effects of the COVID- 19, this study examined trends in maternal syphilis and its impact on congenital syphilis and infant birth outcomes in South Brazil. From a public health standpoint, the negative effect of the COVID- 19 pandemic on the gestational syphilis epidemic in south Brazil is evident, necessitating a refocus on maternal and child health priorities now that the urgency of the pandemic has lessened. Comprehensive strategies are needed to strengthen healthcare systems, expand access to prenatal care and syphilis screening, and ensure timely treatment of pregnant patients with syphilis, thus mitigating the long-term impact of this disease on maternal and child health outcomes. As the world transitions from a crisis response to recovery, prioritizing these essential public health interventions will be crucial to building a resilient healthcare system capable of addressing both ongoing and emerging maternal and child health challenges.

## Data Availability

The data that support the findings of this study are not openly available due to reasons of sensitivity and are available from the corresponding author upon reasonable request. Data are located in controlled access data storage at Hospital Nossa Senhora da Conceição, Porto Alegre, Brazil.

## Abbreviations

WHO: World Health Organization
SUS: Brazilian Unified Health System
SSg: Syphilis status during gestation
VDRL: Venereal Disease Research Laboratory
SSd: Syphilis status at delivery
CST: Congenital syphilis transmission
CDC: Centers for Disease Control
TMSP: Total maternal syphilis prevalence
